# *Pseudomonas aeruginosa* Bacterioferritin Is Assembled from FtnA and BfrB Subunits with the Relative Proportions Dependent on the Environmental Oxygen Availability

**DOI:** 10.3390/biom12030366

**Published:** 2022-02-25

**Authors:** Huili Yao, Anabel Soldano, Leo Fontenot, Fabrizio Donnarumma, Scott Lovell, Josephine R. Chandler, Mario Rivera

**Affiliations:** 1 Department of Chemistry, Louisiana State University, 232 Choppin Hall, Baton Rouge, LA 70803, USA; hyao@lsu.edu (H.Y.); asoldano@lsu.edu (A.S.); lfont39@lsu.edu (L.F.); fabrizio@lsu.edu (F.D.); 2 Protein Structure and X-ray Crystallography Laboratory, University of Kansas, 2030 Becker Dr., Lawrence, KS 66047, USA; swlovell@ku.edu; 3 Department of Molecular Biosciences, University of Kansas, 1220 Sunnyside Ave., Lawrence, KS 66045, USA; jrchandler@ku.edu

**Keywords:** ferritin, bacterioferritin, aerobic culture, microaerophilic culture, anaerobic culture, iron metabolism, iron storage

## Abstract

Ferritins are iron storage proteins assembled from 24 subunits into a spherical and hollow structure. The genomes of many bacteria harbor genes encoding two types of ferritin-like proteins, the bacterial ferritins (Ftn) and the bacterioferritins (Bfr), which bind heme. The genome of *P. aeruginosa* PAO1 (like the genomes of many bacteria) contains genes coding for two different types of ferritin-like molecules, *ftnA* (PA4235) and *bfrB* (PA3531). The reasons for requiring the presence of two distinct types of iron storage protein in bacterial cells have remained largely unexplained. Attempts to understand this issue in *P. aeruginosa* through the recombinant expression of the *ftnA* and *bfrB* genes in *E. coli* host cells, coupled to the biochemical and structural characterization of the recombinant 24-mer FtnA and 24-mer BfrB molecules, have shown that each of the recombinant molecules can form an Fe^3+^-mineral core. These observations led to the suggestion that 24-mer FtnA and 24-mer BfrB molecules coexist in *P. aeruginosa* cells where they share iron storage responsibilities. Herein, we demonstrate that *P. aeruginosa* utilizes a single heterooligomeric 24-mer Bfr assembled from FtnA and BfrB subunits. The relative content of the FtnA and BfrB subunits in Bfr depends on the O_2_ availability during cell culture, such that Bfr isolated from aerobically cultured *P. aeruginosa* is assembled from a majority of BfrB subunits. In contrast, when the cells are cultured in O_2_-limiting conditions, the proportion of FtnA subunits in the isolated Bfr increases significantly and can become the most abundant subunit. Despite the variability in the subunit composition of Bfr, the 24-mer assembly is consistently arranged from FtnA subunit dimers devoid of heme and BfrB subunit dimers each containing a heme molecule.

## 1. Introduction

Iron homeostasis in bacteria (uptake, storage, and utilization) is highly regulated to ensure iron sufficiency while averting Fe^2+^ toxicity [1]. Ferritins are particular to iron homeostasis because they utilize O_2_ or H_2_O_2_ to oxidize Fe^2+^ and compartmentalize the resultant Fe^3+^ in their interior cavity. Hence, ferritins protect cells by enabling the accumulation of iron at concentrations much higher than the solubility of Fe^3+^ while minimizing toxicity caused by the extreme insolubility of the Fe^3+^ ion at physiological pH and the oxidative stress mediated by reactive oxygen species (ROS) resulting from uncontrolled Fe^2+^/Fe^3+^ redox cycling [2,3]. 

The Ferritin family is comprised of three subfamilies: the classical ferritins (Ftn), the bacterioferritins (Bfr), and the DNA binding proteins from starved cells (Dps) [4]. Among these, Ftn and Bfr are typically recognized as iron storage proteins. Ftn are present in animals, plants, fungi, archaea, and bacteria, whereas the Bfr are present only in bacteria and archaea [5,6]. The Bfr and Ftn proteins are nearly spherical, hollow structures assembled from 24 subunits (~12 nm outer diameter and ~8 nm inner diameter). The Bfr exhibit the unique property of binding heme, which is sequestered at the interface of inter-subunit dimers with the heme iron axially coordinated by a conserved methionine (Met52) from each subunit, such that Bfr can bind up to 12 heme molecules [2,7]. The heme functions to mediate electrons from the surface to the compartmentalized Fe^3+^ mineral in order to enable mobilization of Fe^2+^ to the cytosol [8,9,10], and electron transfer from heme to ferroxidase centers may enable H_2_O_2_ detoxification [11]. Ftn and Bfr share an identical subunit fold consisting of a four-helix bundle and a fifth short C-terminal helix nearly perpendicular to the helix bundle. Irrespective of their similar fold and quaternary structures, Bfr and Ftn share low sequence similarity [5,12]. Both, Bfr and Ftn catalyze the oxidation of Fe^2+^ to Fe^3+^ within ferroxidase centers located in the middle of a four-helix bundle that are comprised of conserved residues contributed by all four helices [6]. 

Iron storage in mammalian cells is performed by mammalian ferritins, which are assembled from two types of subunits (H and L). The H subunits harbor a ferroxidase center, whereas the L subunits, which lack ferroxidase centers, are ferroxidase inactive [13]. Bacterial genomes have genes coding for Ftn and Bfr [6]. In a few known cases, such as in the genomes of *Neisseria gonorrhoeae* [14], *Magnetospirillum magnetotacticum* [15], and *Magnetospirillum gryphiswaldense* [16], two distinct genes, each coding for a ferritin, are organized in putative bicistronic operons. Hence, the ferritins in these organisms are thought to assemble from two non-identical subunits. Recently, it has been shown that the bicistronic operon in *M. gryphiswaldense* encodes a Bfr1 subunit that harbors a ferroxidase center but lacks the conserved Met52 residue required for heme binding, and a Bfr2 subunit that contains the conserved Met52 but lacks a ferroxidase center. Bfr1 and Bfr2 were recombinantly expressed in *E. coli* and shown to assemble into a single heterooligomeric 24-mer Bfr similar to the heterooligomeric mammalian Ftn assembled from H and L subunits [16].

In most bacteria with characterized ferritin-like proteins (typified by *E. coli*, fluorescent *Pseudomonas*, and cyanobacteria), the genes coding for Bfr and Ftn are dispersed in the corresponding genomes. Moreover, the bacterial Ftn and Bfr structures of these bacteria are thought to be assemblies of a single type of subunit, such as 24-mer Ftn or 24-mer Bfr. Pioneering work conducted with Bfr isolated from *P. aeruginosa* suggested that the protein could be assembled from two different types of subunit [17]. Subsequent investigations with *P. aeruginosa* PAO1 showed the presence of two distinct genes coding for bacterioferritin, *bfrA* (PA4235) and *bfrB* (PA3531) [18]. 

**Figure 1:**
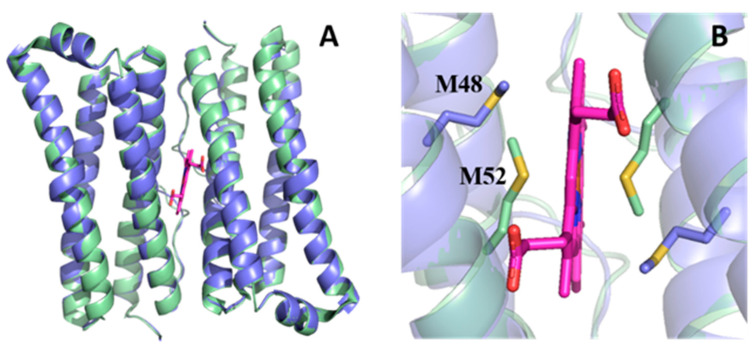
Structural comparison of recombinantly expressed BfrB (PDB ID 5D8O) and FtnA (PDB ID 3R2K) proteins. (**A**) Superposed subunit dimers of BfrB (pale green) and FtnA (slate blue) viewed along the two-fold axis of symmetry; the heme in the BfrB subunit dimer is in magenta. (**B**) Zoomed-in view of the heme, which, in a BfrB subunit dimer, is axially coordinated by Met52 from each subunit (sulfur atoms in yellow). Met48 in the FtnA subunits is too far from the heme to form axial bonds with the heme iron.

Amino acid sequence alignments showed that Met52, the conserved heme ligand in Bfr, is absent in the product of the *bfrA* gene [10]. Recombinant expression of the *bfrA* and *bfrB* genes in *E. coli* host cells followed by structural characterization of the recombinant proteins demonstrated that the products of each of the genes produce 24-mer proteins [19,20]. A major distinguishing factor was that the protein coded by the *bfrB* gene binds heme, whereas the product of the *bfrA* gene does not. Hence, the product of the recombinant *bfrB* gene is a bacterioferritin (BfrB), and the conserved Met52 of each subunit is ideally positioned to coordinate the heme iron (Figure 1) [19]. In contrast, the product of the *bfrA* gene is a ferritin, which cannot bind heme because the closest methionine to the heme (Met48) is too far to coordinate the heme-iron (Figure 1B) [20]. Consequently, gene PA4235 in PAO1 is now annotated as *ftnA*. 

Structural and biochemical characterization of the recombinant 24-mer FtnA and 24-mer BfrB proteins in vitro showed that each have a ferroxidase active site in each subunit, and can accumulate Fe^3+^ in the interior cavity [19,20]. These observations, which lead to the idea that two ferritin-like molecules (FtnA and BfrB) coexist in *P. aeruginosa* cells [20], prompted additional studies to understand the contributions from FtnA and BfrB to iron accumulation in *P. aeruginosa* cells. Studies with cell lysate solutions showed that iron accumulated in BfrB is readily detected, whereas iron accumulated in FtnA is not, even if the cells were cultured in iron rich media. These observations led to the suggestion that 24-mer BfrB functions as the main iron storage protein in *P. aeruginosa* [9]. In this report, we present evidence demonstrating that both FtnA and BfrB play a significant role in iron storage in *P. aeruginosa* under certain conditions because the organism can utilize a heterooligomeric bacterioferritin composed of both FtnA and BfrB subunits. The proportion of FtnA and BfrB subunits constituting a 24-mer bacterioferritin (Bfr) molecule depends on the relative availability of O_2_ in the cell culture. When *P. aeruginosa* cells are cultured in atmospheric air the content of FtnA varies from 0 to 20%, and when the cells are cultured in O_2_-limiting conditions, the content of FtnA subunits in Bfr increases to encompass a range from ~30% to 70%. 

## 2. Materials and Methods

### 2.1. Chemicals, Bacterial Strains, and Culture Media

Chemicals were purchased from Fisher Scientific (Waltman, MA, USA) unless otherwise indicated. *P. aeruginosa* PAO1 was purchased from the University of Washington Genome center (Seattle, WA, USA). The PAO1-derived strain with an unmarked, in-frame deletion of the *bfrB* gene (Δ*bfrB*) was prepared previously [9]. The PAO1-derived strain with an unmarked, in-frame deletion of the *ftnA* gene (Δ*ftnA*) was prepared using the methods reported for the construction of the Δ*bfrB* mutant [9]. In brief: The pEXG2 plasmid [21] used to construct the *ftnA* deletion mutant was made using PCR-amplified DNA products flanking the gene. The resultant PEXG2 derivative was transformed into *E. coli* S17-1 (*λpir*^+^) and then crossed into *P. aeruginosa* PAO1 by mating. Transconjugants were selected on PIA plates containing gentamicin (60 µg/mL), and deletion mutants were selected in no-salt LB agar containing 10% (wt/vol) sucrose. All strains are maintained on Pseudomonas Isolation Agar (PIA, BD Biosciences, San Jose, CA, USA). Cells grown for the isolation of bacterioferritin were cultured in LB media supplemented with 30 µM iron from a 10 mM stock of (NH_4_)_2_Fe(SO_4_)_2_ in water acidified with HCl (pH ~2).

### 2.2. Cell Culture under Aerobic or O_2_-Limiting Conditions

A single colony of *P. aeruginosa* or Δ*ftnA* mutant was used to inoculate starter cultures, which were shake-incubated (220 rpm) for 14 h at 37 °C in 125 mL Erlenmeyer flasks containing 30 mL LB supplemented with 10 μM Fe. When the cells were grown aerobically, the starter culture was used to inoculate six Fernbach flasks containing 1 L of LB media supplemented with 30 μM Fe to an optical density at 600 nm of 0.03. The cells were then cultured at 37 °C for 24 h with shaking (220 rpm), collected by centrifugation (15 min) at 4 °C and 4500 rpm (4392× *g*), then frozen at −80 °C. 

When the cells were grown under microaerobic conditions (10% O_2_ or 2% O_2_) or anaerobically, the starter culture was used to inoculate six 1 L bottles containing 1 L of fresh LB supplemented with 30 µM Fe and 100 mM NaNO_3_. The bottles were sealed with an oxygen permeable membrane and then placed inside a Coy O_2_ Control Glove Box, which allows regulation of O_2_ in 0.1% increments, and the cells were cultured at 37 °C with gentle shaking (30 rpm) for 24 h. 

When the cultures were grown anaerobically, the 1 L bottles were capped, and the oxygen concentration was set at 0.1%. Prior to harvesting cells, the capped bottles were cooled in an ice bath for 30 min. The chilled cell suspensions were transferred to 1 L centrifuge bottles, collected by centrifugation (15 min) at 4 °C and 4500 rpm (4392× *g*), and then frozen at −80 °C. 

### 2.3. Purification of Bacterioferritin (Bfr)

Frozen cell pellets were thawed at room temperature and then suspended in lysis buffer (3 mL/g cell). Lysis buffer consists of 25 mM phosphate buffer (0.4559 g/L KH_2_PO_4_ (Sigma-Aldrich, St. Louis, MO, USA) and 3.771 g/L K_2_HPO_4_ (Sigma-Aldrich, St. Louis, MO, USA), pH 7.5, 10% glycerol, 100 mM NaCl, 1 mM MgSO_4_, 0.5 mM PMSF (VWR, Radnor, PA, USA), 2 mg/mL lysozyme (GoldBio, St. Louis, MO, USA), 0.5% Triton-X100 (Sigma-Aldrich, St. Louis, MO, USA), 0.1 mg/mL DNase (GoldBio, St. Louis, MO, USA), 1 mM TCEP (GoldBio, St. Louis, MO, USA), and 1 tablet Pierce^TM^ protease inhibitor (mini, EDTA-free). 

The cell suspension was stirred at room temperature (20 min), then at 37 °C (30 min), and then sonicated for 20 min in an ice bath with the aid of a Qsonica Q500 sonicator operating with a 45% pulse amplitude and cycles of pulse-on (10 s) and pulse-off (50 s). The cell lysate was clarified by centrifugation (20,500 rpm (50,273× *g*), for 45 min, 4 °C), dialyzed against 4 L of 50 mM Tris (pH 7.6) for 3 h, and then vacuum-filtered through a 0.45 µm membrane filter (VWR, Radnor, PA, USA). The resultant solution was loaded onto a laboratory-packed column (26 mm in diameter) containing ~30 mL of Q Sepharose Fast Flow resin (Sigma-Aldrich, St. Louis, MO, USA) equilibrated with buffer A (50 mM Tris, 1 mM TCEP, and pH 7.6). The column was washed with five column volumes (CV) of buffer A and then eluted with buffer A and a gradient 0–600 mM NaCl. The eluant was fractionated, and the fractions were screened using a Ferene S assay [22] and SDS-PAGE. The Ferene S assay was conducted by mixing 200 µL sample and 10 µL Ferene S reagent (1.1 g ascorbic acid, 1.2 g ammonium acetate, 10 mg neocupronine, and 10 mg Ferene S in water to a final volume of 3 mL). Iron and Ferene S form a blue complex, and because iron can be substantially accumulated in ferritin-like molecules, fractions containing these molecules develop a distinctive blue color within 15 min. These fractions were pooled, and the resultant solution subjected to buffer exchange by three cycles of ultrafiltration (50 kDa, Amicon^®^ Ultra-15 centrifugal filter) and dilution with buffer B (50 mM Tris, 5 mM potassium phosphate, 1 mM TCEP, and pH 7.6). The solution was then loaded onto a hydroxyapatite column (Bio-Scale Mini CHT Type 1, 40 µm cartridge, BIO-RAD, Hercules, CA, USA) equilibrated with buffer B. The column was washed with buffer B (5 CV) and then eluted (20 CV) with buffer B and a gradient of 5–800 mM phosphate. The fractions were screened with the Ferene S assay and SDS-PAGE, and the fractions of interest pooled and then concentrated to ~250 to 500 μL prior to loading into a size exclusion column (Superdex 200 Increase 10/300 GL) equilibrated and eluted with 100 mM phosphate buffer pH 7.6 containing 1 mM TCEP. Fractions eluting from this column contain only Bfr as indicated by a single band in SDS-PAGE experiments.

### 2.4. Determination of BfrB and FtnA Content in Bacterioferritin Samples Isolated from P. aeruginosa Cells

Purified Bfr was exchanged into buffer C (20 mM Tris, 10 mM potassium phosphate, 1 mM TCEP, and pH 7.6) prior to loading onto a high-resolution ion exchange column (Source^TM^ 15Q 4.6/100PE) equilibrated with buffer C. The column was washed with buffer C (5 CV) and eluted (20 CV) with buffer C and a gradient 50–500 mM NaCl. Fractions containing Bfr eluting from the Source^TM^ 15Q 4.6/100PE column were collected and then analyzed by liquid chromatography-mass spectrometry (LC-MS). To this end, Bfr solutions (500 µL) were buffer-exchanged (three cycles) into 10 mM ammonium bicarbonate (pH 7.8) containing 1 mM TCEP using Amicon Ultra filtration units. The resultant solutions were diluted in 0.1% formic acid in water to a final protein concentration of ~1 µM and then incubated at room temperature for 15 min. Determination of the content of FtnA and BfrB was performed with the aid of a Dionex Ultimate 3000 LC (Thermo Scientific, Walthman, MA, USA) coupled to a Bruker Amazon Speed ETD Ion Trap mass spectrometer (Bruker, Billerica, MA, USA). Samples (5 µL) were separated at 60 °C and 0.4 mL/min in a Poroshell 120 EC-C8 column (2.0 × 100 mm, 2.7 µm particle size, 120 Å pore size; Agilent, Santa Clara, CA, USA). Mobile phase (MP) composition: A: 0.1% formic acid in water; B: 0.1% formic acid in acetonitrile; initial conditions 5% B. Following sample injection, the column was eluted with a gradient: hold 5% B (2 min), increase to 50% B (19 min), increase to 90% B (2 min), hold at 90% B (4 min), return to initial conditions (1 min), and equilibrate for 6 min. The ion trap was operated in positive UltraScan mode with electrospray voltage 4500 V and endplate voltage 500 V. The nebulizer was at 35 psi (N_2_), the dry N_2_ gas flow was 10 L/min, and the drying temperature was 250 °C. Spectra were acquired with a maximum accumulation time of 50 ms and ion charge control (ICC) = 250,000. Proteins were detected using full scan mode (400–2000 *m*/*z*), averaging five transient spectra in both profile and line (centroid) mode. LC-MS data were processed and analyzed using Bruker Compass DataAnalysis 4.0. The mass spectra deconvolution tool was used to automate calculation of the molecular mass of components in the mixture. Charge deconvolution was performed over a range 5–30 kDa with a 10% abundance cutoff. Extracted ion chromatograms were generated for the subunits of BfrB (18,552 Da) and FtnA (17,940 Da) using the *m*/*z* values from the three most abundant charge states in the deconvolution, followed by application of a three-point Savitzky–Golay filter to smooth the reconstructed spectra. The peaks of interest were integrated and the relative percentage of FtnA in the sample was calculated with Equation (1).

(1) %FtnA=(FtnA peak area)/(FtnA peak area+BfrB peak area)

### 2.5. Native PAGE

*P. aeruginosa* cells were cultured in 125 mL Erlenmeyer flask containing 15 mL LB media supplemented with 30 μM Fe. Aerobically cultured cells were shake-incubated (220 rpm) for 24 h at 37 °C. Microaerobic (10% O_2_ and 2% O_2_) and anaerobic cultures grown in the presence of 100 mM NaNO_3_ were performed in the O_2_ control glove box with gentle shaking (30 rpm) at 37 °C for 24 h. The cultures were removed from the glove box, cooled in an ice bath (30 min), and centrifuged for 15 min at 4000 rpm (3148× *g*) and 4 °C. 

Cell pellets were resuspended in 1 mL PBS, transferred to 1.5 mL microcentrifuge tubes, and centrifuged for 10 min at 12,500 rpm (15,000× *g*) and 4 °C prior to freezing by immersion in liquid nitrogen. The frozen cells were lysed by addition of 500 μL lysis buffer (50 mM Tris-HCl, pH 8.0, 100 mM NaCl, 1% (*v*/*v*) Triton-X100, 20 mg/mL lysozyme, 0.2 mg/mL DNase, and 1 mM MgSO_4_), followed by three freeze-thaw cycles, and then incubation at 37 °C (10 min) and at ambient temperature (10 min) in a rocker shaker. 

Imaging of iron stored in BfrB and/or FtnA was conducted as previously described [9]: lysate suspensions were centrifuged at 12,500 rpm (15,000× *g*) for 15 min, mixed with 10 μL loading dye (5.9 mL deionized water, 0.5 mL glycerol, 0.4 mL β-mercaptoethanol, 0.4 mL 1% (*w*/*v*) bromophenol blue, and 0.5 mL 1 M Tris-HCl (pH 6.8)), and loaded onto 1.5 mm-thick native PAGE gels (4% stacking and 7.5% resolving gel). Electrophoresis was conducted at 60 V and 4 °C for 9 h, and gels were stained for 10 min in the dark by immersion in 100 mL deionized water containing 0.049 g Ferene S, 250 μL thioglycolic acid and 2.4 mL acetic acid. When appropriate, the gels were destained in 100 mL deionized water and then stained with Coomassie blue.

### 2.6. In-Gel Digestion and Mass Spectrometric Characterization of Proteins

The identification of native bacterioferritin present in *P. aeruginosa* cells was performed by gel LC-MS. Cell culture*,* lysis, native PAGE separation, and gel staining with Ferene S was performed as described above (Native PAGE). Ferene S-stained bands with electrophoretic mobility similar that of recombinant BfrB or FtnA were excised from the gel with a razor blade, transferred to microcentrifuge tubes containing 500 µL acetonitrile (ACN), and incubated (10 min) at ambient temperature. The resultant mix was centrifuged for 1 min (12,500 rpm at room temperature) and the liquid was removed. In-gel reduction of proteins was conducted by the addition of 50 µL 10 mM DTT in 100 mM ammonium bicarbonate buffer at pH 8.0 (ABC) followed by 30 min incubation at 56 °C. The samples were cooled to room temperature prior to the addition of 500 µL ACN, incubation (10 min) at room temperature, centrifugation, and removal of the liquid phase. 

Alkylation of the proteins was conducted by addition of 50 µL 55 mM iodoacetamide in 100 mM ABC, incubation in the dark (20 min, room temp), the subsequent addition of 500 µL ACN, additional incubation (10 min) at room temp, centrifugation, and removal of the liquid phase. The gel slices were then covered with a trypsin solution (13 ng/µL in 10 mM ABC containing 10% *v*/*v* ACN), incubated (90 min) at 4 °C, and then overnight at 37 °C. The resultant peptides were removed from the gel by addition of 100 µL extraction buffer (1:2 *v*/*v* 5% formic acid: ACN), vortexing (2 min), and incubation (15 min) at 37 °C in a shaker. The resulting supernatant was transferred to a new microcentrifuge tube, frozen at −80 °C, and vacuum dried in a SpeedVac (Thermo SAVANT, SPD111V). Dried extracts were dissolved in 50 µL 0.1% formic acid for LC-MS analysis in a Dionex Ultimate 3000 RSLC system (Thermo Scientific, Walthman, MA, USA) coupled to a Q-Exactive Orbitrap spectrometer (Thermo Scientific, Walthman, MA, USA). The mobile phase composition of A and B was as described above. Samples (1 µg) were loaded onto a trap column (C18 PepMap 100, 5 μm, and 100 Å) at 20 μL/min using 2% B. 

Peptides were then separated in an analytical column (Acclaim PepMap 100 (C18), 3 µm, 100 Å) at 0.3 µL/min using a gradient: hold at 5% B (7 min), increase to 10% B (1 min), increase to 25% B (14 min), increase to 90% B (1 min), hold at 90% B (5 min), return to 5% B (1 min), and equilibrate (4 min). Nano electrospray ionization was performed with a stainless-steel emitter (50 µm ID) held at 2.0 kV. Spectra were acquired in data dependent mode with a loop count of 10. Full scans and MS2 scans were acquired with resolutions of 70,000 and 17,500, respectively, isolating ions in a 1.6 *m*/*z* window with normalized collision energy = 28. Raw files were analyzed using Proteome Discoverer (version 2.4, Thermo Scientific) searching with SEQUEST HT (Thermo Scientific, Walthman, MA, USA) and Mascot (Matrix Science, London, UK). Data files were searched against the Uniprot reference proteomes for *P. aeruginosa* (strain ATCC 15692/DSM 22644/CIP 104116/JCM 14847/LMG 12228/1C/PRS 101/PAO1), proteome ID UP000002438).

### 2.7. Measurement of K_d_ by Surface Plasmon Resonance (SPR)

SPR experiments were performed at 25 °C using a Biacore X100 instrument (GE Healthcare, Chicago, IL, USA) and a previously reported protocol [23,24]. In brief: bacterioferritin was immobilized on CM5 sensor chips (Cytiva, Marlborough, MA, USA) using amine coupling chemistry. The sensor surface was conditioned with 50 mM NaOH, 10 mM HCl, 0.1% SDS, and 0.085% (*w*/*v*) H_3_PO_4_, and then activated with 0.1 M N-hydroxysuccinimide (NHS) and 0.5 M N-ethyl-N′-3-(dimethylaminopropyl)carbodiimide hydrochloride (EDC) in water. 

The immobilization of Bfr or FtnA_rec_ at the activated surface was performed by flowing (5 µL/min, 600 s) a solution of Bfr or FtnA_rec_ (100 nM) in 10 mM sodium acetate buffer (pH 5.0). Activated sites not bound to Bfr were quenched by flowing (10 µL/min for 420 s) 1.0 M aqueous ethanolamine-HCl (pH 8.5). A flow cell activated by EDC/NHS and quenched by ethanolamine was used as the reference surface. To measure *K*_d_ values, a solution of Bfd in SPR buffer (PBS pH 7.4 with 1 mM TCEP) was simultaneously passed (5 µL/min) over the cell with immobilized Bfr or FtnA_rec_, allowing for 120 s contact time and 120 s dissociation time. 

Corrected SPR responses were obtained by subtracting the response of the reference cell, and a binding curve was constructed using the corrected SPR responses obtained from flowing Bfd at the concentrations specified in Figure 8. Dissociation constants (*K*_d_) were evaluated as described in the Biacore Evaluation Software Handbook using Equation (2), where *R_eq_* is the SPR response at the plateau when the system reaches steady state equilibrium; *R_max_* is the theoretical binding capacity (also known as the maximum SPR response caused by binding of the analyte (Bfd) to the immobilized protein (Bfr or FtnA)); [*Bfd_f_*] is the concentration of Bfd passed over immobilized Bfr or FtnA; *K*_d_ is the dissociation constant, *n*, which is the steric interference factor specifying the number of binding sites in Bfr blocked by binding one analyte molecule (Bfd), was set to 0.91.

(2) $$R_{eq}=R_{max}[Bfd_{f}]/(K_{d}+n[Bfd_{f}])$$

## 3. Results and Discussion

### 3.1. P. aeruginosa Bacterioferritin (Bfr) Is a Heterooligomeric Molecule Constituted by FtnA and BfrB Subunits

In previous studies, we showed that the recombinant expression of *P. aeruginosa* PAO1 genes PA4235 (*ftnA*) or PA3531(*bfrB*) in *E. coli* host cells enables isolation of recombinant 24-mer FtnA or 24-mer BfrB proteins [19,20]. These observations, which suggested that FtnA and BfrB molecules may coexist in the *P. aeruginosa* cell, led us to ask whether FtnA and BfrB serve similar or different roles in iron metabolism, with the long-term goal of understanding why the genomes of *Pseudomonas* and other bacteria harbor genes coding for ferritin and bacterioferritin. To investigate the relative contributions of FtnA and BfrB to intracellular iron storage, we constructed *P. aeruginosa* strains with unmarked, in-frame deletions of the *bfrB* (Δ*bfrB*) or *ftnA* (Δ*ftnA*) genes. 

**Figure 2:**
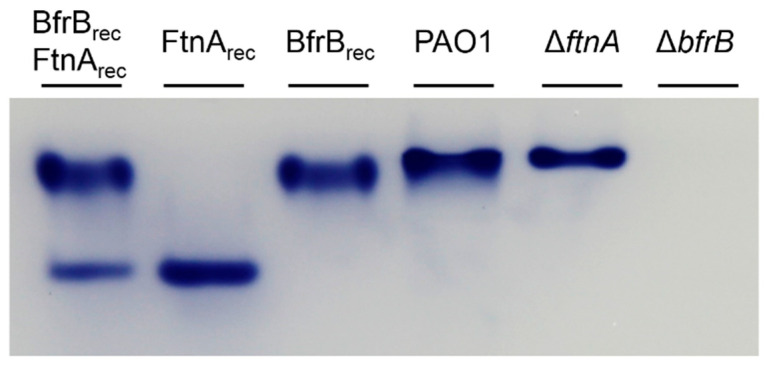
Iron stored in ferritin-like molecules visualized in native polyacrylamide gels stained with Ferene S. BfrB_rec_ and FtnA_rec_ were used as electrophoretic mobility standards, loaded as a mixture or alone. Lysates of PAO1 and Δ*ftnA* cells grown aerobically for 24 h in LB media supplemented with 30 µM Fe show an iron-stained band matching that of BfrB_rec_, whereas lysates of Δ*bfrB* cells cultured similarly are characterized by the conspicuous absence of accumulated iron.

The PAO1, Δ*bfrB*, and Δ*ftnA* strains were cultured aerobically in LB media supplemented with 30 µM iron. To visualize iron stored in BfrB and FtnA, we harvested and lysed the cells from each of the cultures, separated the cell lysate solutions in native polyacrylamide gels, and then stained the gels with Ferene S, which forms a blue complex with Fe^2+^ and allows the imaging of iron accumulated in ferritin-like molecules. As shown in Figure 2, recombinant FtnA (FtnA_rec_) and recombinant BfrB (BfrB_rec_) exhibit different electrophoretic mobility, independent of having been loaded onto the gel as either pure protein or as a mixture of FtnA_rec_ and BfrB_rec_. 

Although the lysate solutions obtained from PAO1 cells cultured aerobically reproducibly produce an iron-stained band matching the electrophoretic mobility of BfrB_rec_, a band corresponding to the electrophoretic mobility of FtnA_rec_ is noticeably absent. These observations were interpreted to suggest that under the culture conditions used in the experiments *P. aeruginosa* cells primarily store iron in BfrB, rather than in FtnA [9]. To further explore this idea, we studied the lysate solutions from Δ*ftnA* and Δ*bfrB* cells with the expectation that the absence of BfrB might “force” iron to be stored in FtnA. We found that, while the cell lysate solutions from Δ*ftnA* cells produced a band with electrophoretic mobility matching that of BfrB_rec_ (Figure 2), the cell lysate solutions obtained from the Δ*bfrB* cells did not produce an iron-stained band, which appears to suggest that the absence of BfrB does not force iron accumulation in FtnA. The intriguing absence of FtnA-stored iron in *P. aeruginosa* is made even more enigmatic when considering previously published in vitro observations, which demonstrated the ability of FtnA_rec_ to form 24-mer molecules with a catalytically active ferroxidase center in each subunit and the ability to store a Fe^3+^ mineral in the interior cavity [20]. 

To unravel the mystery, we focused the current study on understanding the role of FtnA in the iron metabolism of *P. aeruginosa*. Initial clues emerged from close inspection of the PAGE experiments summarized in Figure 2. The iron-stained band observed in the Δ*ftnA* cell lysates is consistently narrower than the band produced by the PAO1 cell lysates. These observations led us to hypothesize that the electrophoretic mobility of BfrB in lysate solutions of PAO1 cells may be influenced by FtnA—for example, by a heterooligomeric Bfr composed of FtnA and BfrB subunits. 

To test this hypothesis, we excised the bands from a native gel and subjected the samples to proteolytic digestion and subsequent analysis by LC-MS. The results show that, as expected, BfrB is present in the band excised from the Δ*ftnA* lysate lane (Table S1), and, consistent with our hypothesis, both BfrB and FtnA are present in the band excised from the PAO1 lysate lane (Table S2). These findings suggest the presence of a heterooligomeric bacterioferritin (Bfr) assembled from FtnA and BfrB subunits. To obtain supporting evidence, we proceeded to isolate native Bfr from *P. aeruginosa* cells and determine the nature of its subunit composition. The results from these experiments are presented below.

To isolate native bacterioferritin, we cultured PAO1 cells aerobically in LB media supplemented with 30 µM iron, shaking the culture flasks at 220 rpm for 24 h. Following cell harvesting and lysis, the clarified supernatant was subjected to a column chromatography workflow that includes sequentially, anion exchange (Q-Sepharose), “pseudo-affinity” (hydroxyapatite), and size exclusion (Superdex-200) columns. Pure protein, as ascertained from SDS-PAGE gels, eluted from the Superdex-200 column as a single peak with an elution volume matching that of a ferritin standard (Figure S1), indicating that the pure protein is a 24-mer bacterioferritin. 

**Figure 3:**
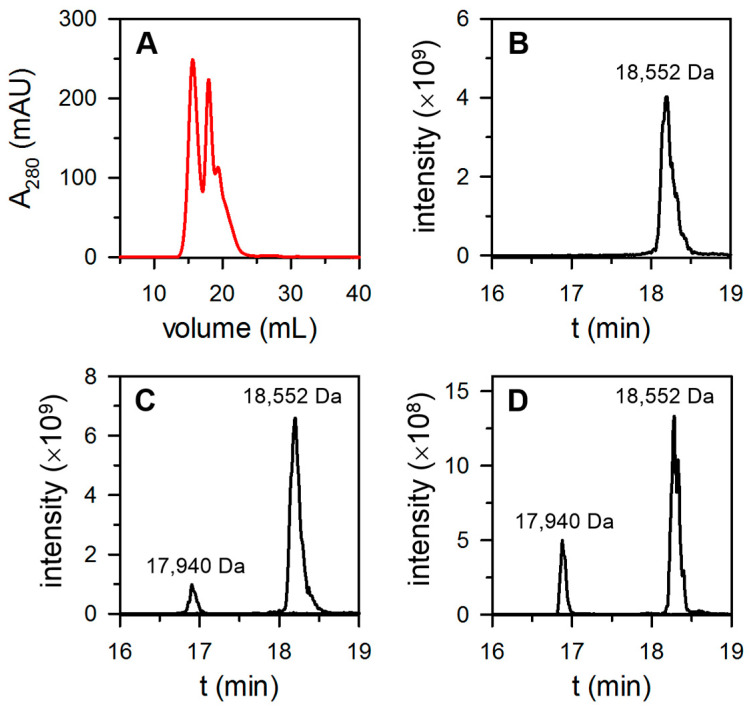
Bfr isolated from aerobically cultured *P. aeruginosa* is a mix of proteins consisting of 24-mer BfrB and 24-mer heterooligomeric Bfr constituted of FtnA and BfrB subunits. (**A**) Pure Bfr injected on a Source^TM^ 15Q column eluted in two peaks and a shoulder. (**B**) The extracted ion chromatogram (EIC) obtained by LC-MS analysis of protein eluted from the Source^TM^ 15Q in the first peak shows only BfrB (18,552 Da). (**C**,**D**) The EICs obtained from LC-MS analysis of protein eluted in the second peak and in the shoulder, respectively, show the presence of FtnA (17,940 Da) and BfrB subunits. The content of FtnA and BfrB subunits in Bfr was estimated from the corresponding integrated peak areas in the EIC, and the values are presented in Table 1.

Surprisingly, when the pure 24-mer bacterioferritin was loaded onto a Source^TM^ 15Q column (high resolution anion exchange), it eluted in two peaks and a shoulder (Figure 3A). Analysis of the eluant by SDS-PAGE shows that protein eluted in the two peaks and shoulder exhibited only one band, which corresponded to a molecular weight of approximately 18 kDa. We hypothesized that the distinct species eluting from the Source^TM^ 15Q column in each of the peaks are Bfr molecules that differ in their content of FtnA and BfrB subunits. 

The relatively small difference in the molecular weights of FtnA and BfrB, 17,940 Da and 18,552 Da, respectively, makes the corresponding bands indistinguishable in SDS-PAGE gels. This difference in molecular mass, however, is readily distinguished in mass spectrometry experiments. Consequently, we sampled an aliquot from each of the three peaks eluting from the Source^TM^ 15Q column, exchanged the buffer to ammonium acetate prior to diluting in 0.1% acetic acid and injected the resultant solution onto a reverse phase column for LC-MS analysis as described in the Materials and Methods section. 

When this process was conducted with a sample from the first peak eluting from the Source^TM^ 15Q column, only one peak was observed in the ion extracted chromatogram (18,552 Da), which corresponded to the molecular mass of a BfrB subunit (Figure 3B). These observations indicate that the bacterioferritin eluting from the Source^TM^ 15Q column in the first peak is assembled only from BfrB subunits. In contrast, LC-MS analysis of the second peak eluting from the Source^TM^ 15Q column produced an ion extracted chromatogram (Figure 3C) with peaks corresponding to the molecular masses of FtnA (17,940 Da) and BfrB (18,552 Da) subunits, respectively. The %FtnA, which was calculated from the integrated areas of the peaks in the extracted-ion chromatogram, indicates that the protein eluting from the Source^TM^ 15Q column in the second peak contains ~10% FtnA. A similar analysis conducted with the protein eluting from the Source^TM^ 15Q in the shoulder (Figure 3D) indicates the presence of Bfr harboring up to 21% FtnA (Table 1). 

### 3.2. The Proportion of FtnA and BfrB Subunits in Heterooligomeric Bfr Depends on Environmental O_2_ Levels in the Bacterial Culture

The *ftnA* (PA4235) and *bfrB* (PA3531) genes are not genetically linked in the genome of *P. aeruginosa* PAO1 and in the genomes of other *Pseudomonas*. The gene adjacent to the *bfrB* gene is the *bfd* gene, which codes for a bacterioferritin-associated ferredoxin. These genes are not associated in an operon and appear to be independently regulated. Transcription of the *bfrB* gene is stimulated by environmental iron availability, whereas the *bfd* gene is stimulated by iron starvation [25]. 

Bfd is a [2Fe-2S] ferredoxin [24], which binds and transfers electrons to the Fe^3+^ stored in Bfr to facilitate mobilization of Fe^2+^ to the cytosol for its incorporation in metabolism [8,9,26,27]. The *ftnA* gene (formerly annotated as *bfrA*), which is adjacent to a gene coding for a heme catalase (*katA*), does not respond to environmental iron levels, and its transcription is independent of *katA* transcription. A more recent report suggests that transcription of *ftnA* is under control of the Anr transcription regulator [28]. 

Anr functions to enable the adaptation of *P. aeruginosa* cells to the microoxic environment of stationary phase planktonic cells and to the microoxic to anoxic continuum characteristic of biofilms [28,29,30]. This adaptation is enabled via transcriptional activation of the Anr regulon, which includes, among others, genes coding for cytochrome c oxidases with high affinity for O_2_ (Cbb_3_-2), O_2_ transport under microoxic conditions (hemerythrin), heme biosynthesis under microoxic/anoxic conditions (HemN and HemF), oxidative stress defense (KatA and CcpR), and Fe^2+^ uptake (FeOABC). 

That the content of FtnA in heterooligomeric Bfr isolated from cells cultured aerobically is in the range of 0 to 20% may be explained in the context of *bfrB* transcription being stimulated by high environmental iron, *ftnA* transcription being stimulated by low environmental O_2_, and the known fact that *P. aeruginosa* cells cultured aerobically block the transfer of O_2_ from the gas to the liquid phase, such that cells in late-exponential and stationary phase experience microoxic conditions [31]. 

Therefore, we suggest that, in the early and mid-exponential phase, when dissolved O_2_ levels in the media are high, the availability of iron (30 µM) stimulates *bfrB* transcription and leads to iron accumulation in 24-mer BfrB molecules. As the cell density increases and the concentration of dissolved O_2_ in the culture media decreases, however, Anr facilitates the transition from aerobic to microaerophilic growth by stimulating transcription of genes in the Anr regulon, which includes *ftnA*. The resultant FtnA subunits assemble into heterooligomeric Bfr molecules that contain ~8‒20% FtnA, which coexist with homooligomeric BfrB molecules. These ideas led us to hypothesize that culturing *P. aeruginosa* under microaerophilic conditions should lead to an increased content of FtnA in Bfr. To test the hypothesis, we cultured *P. aeruginosa* cells in a Coy O_2_ Control Glove Box, which allows for the regulation of O_2_ in 0.1% increments. The results from these studies are presented below.

**Figure 4:**
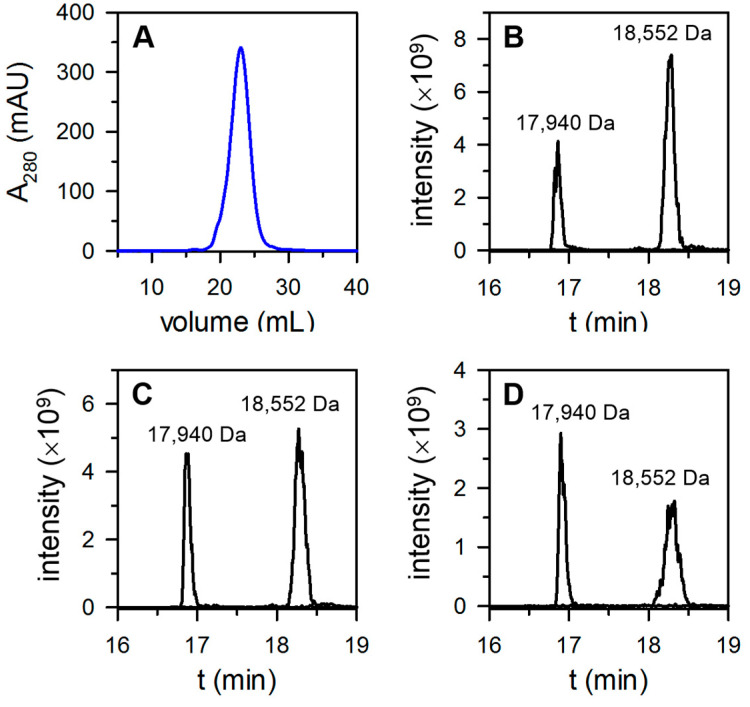
Bfr isolated from *P. aeruginosa* cultured in 10% O_2_ is a mix of 24-mer heterooligomeric protein constituted of FtnA and BfrB subunits of varying content. (**A**) Pure Bfr injected on a Source^TM^ 15Q column eluted in a single, broad peak. The extracted ion chromatograms (EICs) obtained by LC-MS analysis of protein eluted from the Source^TM^ 15Q column in the left (**B**), center (**C**), and right (**D**) portions of the broad peak show the presence of FtnA (17,940 Da) and BfrB (18,552 Da) subunits. The content of FtnA and BfrB in Bfr was estimated from the corresponding integrated peak areas in the EIC, and the values are presented in Table 1.

Bfr from *P. aeruginosa* cells cultured under low O_2_ (10% O_2_ or 2% O_2_) was purified to homogeneity using the column workflow described above. Pure Bfr eluted from the Superdex-200 column in a single peak with a retention volume corresponding to a 24-mer assembly (Figure S1). The 24-mer bacterioferritin purified from cells cultured in 10% O_2_ eluted from a Source^TM^ 15Q as a single, relatively broad peak (Figure 4A). 

To determine the content of FtnA and BfrB subunits, three fractions were sampled from the peak corresponding to the early-, middle-, and late-eluting sections for subsequent LC-MS analysis. Figure 4B,C depicts the corresponding ion extracted chromatograms, which indicate the presence of FtnA (17,940 Da) and BfrB (18,552 Da) subunits. Inspection of the chromatograms suggests that the content of FtnA in Bfr is lower in the molecules eluting from the Source 15Q column in the early fractions and increases in the molecules eluting in the late fractions. Integration of the EIC peaks indicates that the content of FtnA in Bfr isolated from cells cultured in the presence of 10% O_2_ ranged from 28% to 49% (Table 1). 

Performing a similar analysis with Bfr isolated from cells cultured in the presence of 2% O_2_ showed that the content of FtnA spanned a range encompassing 30% to 59% (Table 1 and Figure S2). These observations, which support the hypothesis that the FtnA content in Bfr increases when *P. aeruginosa* is cultured under microaerophilic conditions, prompted us to investigate the composition of Bfr isolated from cells cultured under anaerobic conditions. 

To culture cells anaerobically, 1 L bottles containing 1 L of LB media were inoculated inside the Coy box equilibrated at the lowest O_2_ setting (0.1% O_2_) and immediately capped, which led to the rapid depletion of residual O_2_ in the culture and subsequent anaerobic growth. Bfr was purified from these cells with the same chromatographic workflow described above. LC-MS analysis showed that the content of FtnA in the isolated Bfr spanned a range from 29% to 69% (Table 1 and Figure S3).

### 3.3. Do 24-mer FtnA Molecules Assemble in P. aeruginosa?

**Figure 5:**
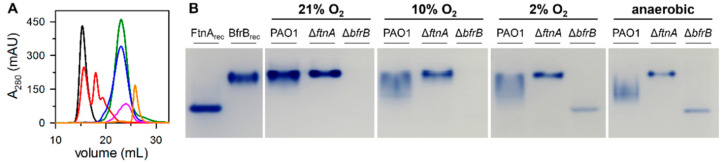
(**A**) Overlay of chromatographic traces from injecting a high-resolution anion exchange column (Source^TM^ 15Q) with pure bacterioferritin solutions isolated from *P. aeruginosa* PAO1 cells cultured aerobically (21% O_2_ red), in 10% O_2_ (blue), in 2% O_2_ (green), and anaerobically (pink). The black trace corresponds to Bfr isolated from Δ*ftnA* cells cultured in 21% O_2_ and the orange trace corresponds to FtnA_rec_. (**B**) Lysate solutions of PAO1, Δ*ftnA* and Δ*bfrB* cells cultured under distinct O_2_ levels in LB media supplemented with 30 μM Fe were separated in a native gel and stained with Ferene S.

The results presented thus far indicate that *P. aeruginosa* stores iron in Bfr, which can be assembled exclusively of BfrB subunits or from FtnA and BfrB subunits. The content of FtnA in Bfr, which has been shown to increase as the O_2_ concentration in the cell culture decreases (Table 1), exerts significant influence on the elution volume (V_e_) of Bfr from a Source^TM^ 15Q column (Figure 5A). The smaller V_e_ corresponds to 24-mer BfrB, which was isolated from Δ*ftnA* cells (black trace) or from aerobically cultured PAO1 cells (red trace, peak 1), and the largest V_e_ is associated with the elution of FtnA_rec_ (orange). These observations indicate a more tenacious association of FtnA subunits with the Source^TM^ 15Q column resin. In agreement, the V_e_ values displayed by the distinct heterooligomeric Bfr proteins become progressively larger as the FtnA content in Bfr increases. In this context, it is interesting to note that the V_e_ associated with the elution of Bfr isolated from anaerobically cultured cells (pink) is smaller than the V_e_ associated with FtnA_rec_. This observation, which is consistent with a maximum 69% FtnA content in Bfr isolated from anaerobically cultured cells (Table 1), underscores the fact that 24-mer FtnA has not been isolated from *P. aeruginosa*, even when the cells were cultured anaerobically. These observations, therefore, suggest that 24-mer FtnA is either not assembled in *P. aeruginosa* under our experimental conditions or alternatively, is present at low levels that prevent its isolation. 

To further explore this issue, we resorted to separating cell lysates in native gels and imaging the iron accumulated in Bfr with the aid of Ferene S staining. The results from these experiments are shown in Figure 5B. As indicated above, the lysate solution from PAO1 cells cultured aerobically produced a broad iron-stained band that closely matches the electrophoretic mobility of BfrB_rec_. When the PAO1 cells were cultured micro aerobically (10% O_2_ or 2% O_2_) or anaerobically, the corresponding iron-stained bands became significantly broader and exhibited electrophoretic mobility intermediate between that characteristic of BfrB_rec_ and FtnA_rec_. These observations are consistent with the idea that the content of FtnA in Bfr increases when *P. aeruginosa* cells are cultured in microaerobic or anaerobic conditions. The PAO1 cell lysates did not produce an iron-stained band with electrophoretic mobility matching that of FtnA_rec_. In comparison, note that the lysate solutions of Δ*bfrB* cells cultured in 2% O_2_ or anaerobically enabled the detection of an iron-stained band matching the electrophoretic mobility of FtnA_rec_. The presence of FtnA in the iron-stained band was corroborated by slicing the band from the gel and submitting the sample to proteolytic digestion and analysis by LC-MS (Table S3). Taken together, these findings support the idea that *P. aeruginosa* cells accumulate iron in bacterioferritin molecules with variable subunit composition. Cells cultured aerobically harbored Bfr molecules whose subunit composition ranged from only BfrB to ~80% BfrB. When cells were cultured in reduced O_2_ conditions, the content of BfrB reduced further, reaching values as low as ~30% in our experiments. The absence of detectable iron-stained bands matching the electrophoretic mobility of FtnA_rec_ in lysates from PAO1 cells strongly suggests that iron does not accumulate in 24-mer FtnA, although iron can accumulate in 24-mer FtnA when the *bfrB* gene has been deleted and the cells are cultured in reduced O_2_ conditions. 

### 3.4. Bfr Is Assembled from FtnA and BfrB Inter-Subunit Dimers

Although the data presented thus far demonstrate that *P. aeruginosa* Bfr is a heterooligomeric molecule composed of FtnA and BfrB subunits, it does not indicate how individual FtnA and BfrB subunits assemble in a 24-mer Bfr. In this section, we present evidence supporting the idea that FtnA inter-subunit dimers and BfrB inter-subunit dimers assemble into 24-mer Bfr. 

**Figure 6:**
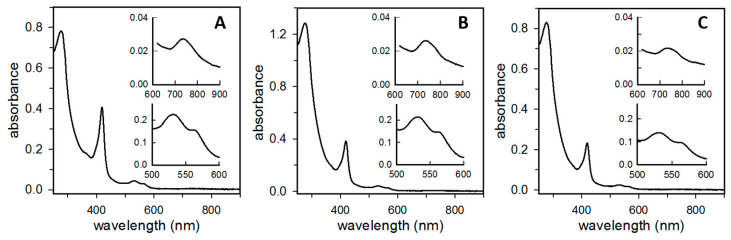
The electronic absorption spectrum of Bfr isolated from *P. aeruginosa* indicates that heme is axially coordinated by two Met52 residues within BfrB inter-subunit dimers. Spectra shown correspond to Bfr isolated from cells cultured (**A**) aerobically, (**B**) micro aerobically in 2% O_2_, and (**C**) anaerobically; the corresponding protein concentrations in mg/mL are 0.7, 0.9, and 0.6. The full spectra were obtained in a 0.2 cm path-length cuvette, and the traces in the insets were measured in a 1.0 cm path-length cuvette. The full spectra highlight the characteristic 418 nm Soret bands and the strong feature with absorption maximum ca. 300 nm, which originated from mineralized Fe^3+^ in the interior cavity. The bottom insets highlight the α- and β-bands (~560 and ~530 nm), and the top insets highlight the weak 740 nm band characteristic of bis-Met heme ligation in bacterioferritin.

The electronic absorption spectra of Bfr isolated from *P. aeruginosa* cells cultured aerobically, micro aerobically, or anaerobically are shown in Figure 6. Note that all the spectra exhibit a Soret band with an absorption maximum at 418 nm; α- and β-bands at 567 and 527 nm, respectively; and a low intensity band ca. 740 nm. Together, these spectral features, which are diagnostic of bacterioferritin-heme axially coordinated by two Met ligands [10,32,33,34], demonstrate that heme in the heterooligomeric Bfr is axially coordinated by two Met residues. As shown in Figure 1, this is only possible in BfrB inter-subunit dimers, where a Met52 ligand from each subunit axially coordinates the heme iron. Hence, the spectral features strongly suggest that the Bfr molecules are assemblies of BfrB inter-subunit dimers, each containing a heme molecule, and FtnA inter-subunit dimers devoid of heme. 

**Figure 7:**
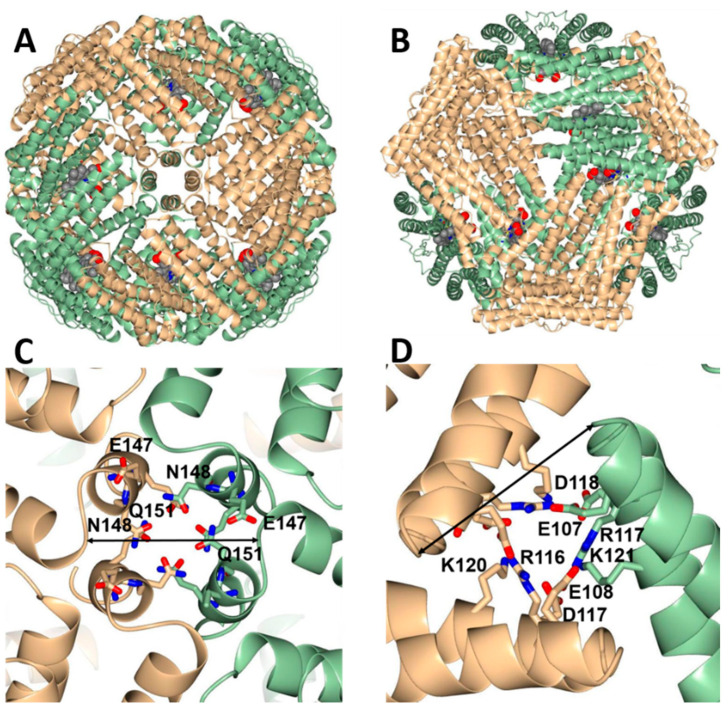
In silico model showing one of the possible arrangements in which six FtnA inter-subunit dimers (wheat) and six BfrB inter-subunit dimers (green) assemble into a 24-mer Bfr. Heme (red) is contained between each BfrB inter-subunit dimer. (**A**,**B**) views of the heterooligomeric 24-mer Bfr viewed along a four-fold and a three-fold pore, respectively. (**C**,**D**) are close-up views of the corresponding three-fold and four-fold pores, which are nearly indistinguishable from the corresponding pores in the structures of FtnA_rec_ (PDB ID 3R2K) and BfrB_rec_ (PDB ID 5D8O) used in the model construction (see text). The distances across the four-fold and three-fold pore are 17 and 16 Å, respectively and are indicated by the arrows in (**C**,**D**).

To further explore this idea, we created an in-silico model of heterooligomeric Bfr comprised of six BfrB inter-subunit dimers and six FtnA inter-subunit dimers (Figure 7). To this end, the 24 subunits in the X-ray crystal structure of FtnA_rec_ were aligned with the 24 subunits in the X-ray structure of BfrB_rec_. The structural alignment shows that the 24-mer structures are nearly identical, with RMSD ~0.7 Å (C_α_) for 3672 residues in the 24 subunits. To create the heterooligomeric Bfr model, six BfrB subunit dimers were removed from the 24-mer BfrB_rec_ structure, and six FtnA subunit dimers from the alignment were merged in place of the deleted BfrB subunit dimers. 

The resultant model shows that inter-subunit dimers of BfrB and inter-subunit dimers of FtnA pack seamlessly into a 24-mer architecture nearly indistinguishable from the structures of recombinant BfrB and FtnA. It is also noteworthy that residues lining the four-fold and three-fold pores are conserved. This results in three-fold and four-fold pores in the heterooligomeric Bfr molecule (Figure 7) structurally identical to those observed in the structures of recombinant 24-mer BfrB or recombinant 24-mer FtnA.

### 3.5. Bfd Binds Heterooligomeric Bfr at BfrB Inter-Subunit Dimers

The X-ray crystal structure of the complex formed by BfrB_rec_ and Bfd, hereafter referred to as the (BfrB-Bfd)_rec_ complex, revealed that up to 12 Bfd molecules can bind at identical sites on the BfrB_rec_ surface, at the interface of each inter-subunit dimer, above a heme molecule [8]. Studies conducted with the (BfrB-Bfd)_rec_ complex showed that heme mediates electron transfer between the [2Fe-2S] cluster of Bfd and the Fe^3+^ mineral in the BfrB_rec_ cavity, promoting the mobilization of Fe^2+^ [8,10]. Measurements conducted with the aid of SPR showed that the 12 Bfd binding sites on BfrB_rec_ are equivalent and independent, where Bfd binds with a *K*_d_ of 3.3 µM [23]. 

We also demonstrated that blocking the bacterioferritin-Bfd complex in *P. aeruginosa* cells by deletion of the *bfd* gene (Δ*bfd*) induces iron homeostasis dysregulation by causing an irreversible accumulation of Fe^3+^ in bacterioferritin and concomitant iron deprivation in the cytosol [9,35], dysregulation of sulfur metabolism, oxidative stress [35], and impaired biofilm maintenance [26]. The structure of the (BfrB-Bfd)_rec_ complex was also used to guide the design of novel small molecules, which bind recombinant BfrB at the Bfd binding site [36]. These small molecules have been shown to penetrate the *P. aeruginosa* cell envelope, bind to bacterioferritin, inhibit iron mobilization from bacterioferritin, and kill biofilm-embedded bacteria [27,36].

**Figure 8:**
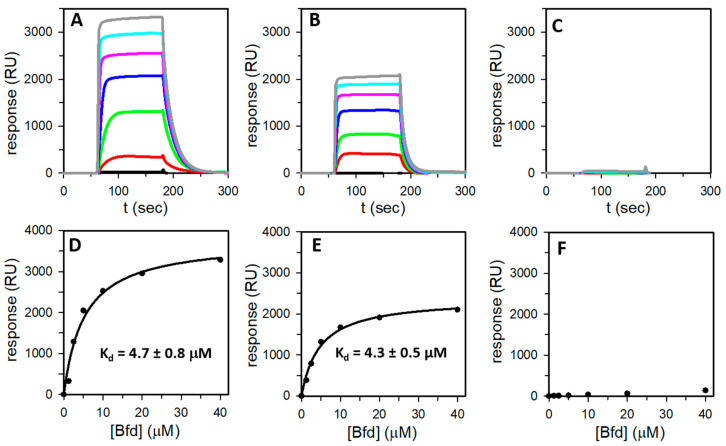
Bfd binds Bfr at the surface of BfrB inter-subunit dimers. The association between Bfr and Bfd was studied with the aid of SPR. Reference and baseline-subtracted responses obtained from flowing Bfd solution over: (**A**) immobilized Bfr containing only BfrB subunits, isolated from cells cultured in 21% O_2_ (see Table 1), (**B**) immobilized Bfr containing 45% FtnA isolated from cells cultured in 2% O_2_, and (**C**) immobilized FtnA_rec_. The concentrations of Bfd are 0 µM (black), 1.25 μM (red), 2.5 µM (green), 5 μM (blue), 10 µM (pink), 20 μM (cyan), and 40 μM (grey). The binding affinity was determined by steady-state analysis. (**D**,**E**) The steady state responses shown in (**A**,**B**), respectively, plotted as a function of Bfd concentration (black circles) and fitted to the binding model (solid line) described by equation 2. (**C**,**F**) Bfd does not bind to FtnA_rec_.

Given the significant role played by the bacterioferritin-Bfd protein–protein interaction in the regulation of iron homeostasis and as potential target for the development of antibiotic and antibiofilm compounds, we studied the association of heterooligomeric Bfr and Bfd with the aid of surface plasmon resonance (SPR). These experiments were conducted with three distinct samples, each immobilized on a CM5 sensor chip: (1) Bfr isolated from PAO1 cells cultured aerobically; specifically, protein that contains only BfrB subunits (see Table 1). (2) Bfr isolated from PAO1 cells cultured in 2% O_2_; specifically, protein that contains ~45% FtnA. (3) FtnA_rec_. Figure 8A–C depict the reference and baseline-subtracted responses obtained when passing Bfd over each of the immobilized Bfr or FtnA_rec_. 

In the experiments conducted with Bfr immobilized on the sensor chip (Figure 8A,B), the response, which increased as a function of Bfd concentration, clearly indicates a specific association between Bfd and Bfr. In contrast, a similar experiment with immobilized FtnA_rec_ indicates that Bfd does not bind to FtnA (Figure 8C). The plateau reached in each of the responses obtained with immobilized Bfr indicate steady state equilibrium and plotting each response at steady state as a function of the Bfd concentration results in the hyperbolic binding curves described by the black circles in Figure 8D,E. Analysis of these curves shows that Bfd binds to Bfr with a *K*_d_ nearly identical to what was previously reported for the association of Bfd to BfrB_rec_ (Table 2). It is also noteworthy that Bfd binds to Bfr with nearly the same *K*_d_ (Table 2) irrespective of the FtnA subunit content in the 24-mer assembly. In this context, it is important to underscore that the experiments with Bfr isolated from cells cultured in 21% O_2_ or 2% O_2_ were conducted with nearly identical immobilization levels of Bfr on the sensor chip (see Table 2), thus, with similar amounts of immobilized Bfr at the surface. Consequently, it is significant that the maximum response (R_max_) obtained with Bfr isolated from cultures in 2% O_2_ (~55% BfrB) is ~60% the R_max_ obtained with Bfr isolated from cultures in 21% O_2_ (100% BfrB) because it supports the idea that Bfd binds Bfr at BfrB inter-subunit dimers, as seen in the X-ray crystal structure of the (BfrB-Bfd)_rec_ complex [8,23]. That Bfd does not bind to FtnA inter-subunit dimers within Bfr is supported by the SPR data, which shows that Bfd does not bind to FtnA_rec_.

## 4. Conclusions

Our findings indicate that the iron storage function in *P. aeruginosa* PAO1 is performed by a heterooligomeric bacterioferritin, where the relative content of FtnA and BfrB subunits is strongly affected by the environmental O_2_ levels. The heterooligomeric Bfr in *P. aeruginosa* is similar to the heterooligomeric mammalian Ftn assembled from H and L subunits. A significant distinguishing factor, however, is that, in Bfr, both types of subunits (FtnA and BfrB) have functional ferroxidase centers. 

Our data also suggests that, when *P. aeruginosa* cells have unrestricted access to environmental O_2_ and iron, the majority of Bfr molecules harbor only BfrB subunits. *P. aeruginosa*, however, are thought to prefer microoxic conditions [37], such that aerobically cultured planktonic cells in the midlogarithmic and stationary phases establish microoxic conditions by blocking O_2_ transfer from the gas to the liquid phase [31], and cells in biofilms experience a continuum of microoxic to anoxic environments. Under these conditions, the Anr regulator stimulates transcription of the *ftnA* gene, which, in turn, leads to the assembly of heterooligomeric Bfr. Despite the stimulatory effect of Anr on *ftnA* gene transcription, iron in *P. aeruginosa* cells does not accumulate in 24-mer FtnA assemblies, even under anaerobic growth conditions. Hence, the existing evidence suggests that *P. aeruginosa* utilizes homooligomeric 24-mer BfrB and heterooligomeric 24-mer (FtnA and BfrB) molecules to store Fe^3+^.

In previous work, we showed that in *P. aeruginosa* bacterioferritin regulates cytosolic iron concentrations by (i) oxidizing Fe^2+^ and storing Fe^3+^ in its internal cavity and (ii) forming a complex with Bfd to reduce Fe^3+^ in the internal cavity and mobilize Fe^2+^ to the cytosol [9,35]. From the perspective of Fe^2+^ oxidation (ferroxidase activity), it is important to underscore that heterooligomeric Bfr molecules have two distinct types of ferroxidase center: one associated with the BfrB subunits and the second associated with the FtnA subunits [2,19,20]. Given that the FtnA content increases significantly when *P. aeruginosa* cells experience microoxic conditions, it is tempting to speculate that the di-Fe^2+^ moiety in the ferroxidase center of FtnA subunits may exhibit a higher affinity for O_2_, which may enable ferroxidase activity under the low intracellular O_2_ levels expected when cells are cultured in microoxic conditions. Along the same vein, the di-Fe^2+^ moiety in the ferroxidase center of BfrB subunits may exhibit a lower affinity for O_2_ but may be tuned to react rapidly with intracellular H_2_O_2_, which can be expected to be present when cells are cultured aerobically, as has been suggested for the ferroxidase center of *E. coli* bacterioferritin [38]. Clearly, the discovery of a mixed subunit Bfr in *P. aeruginosa* brings additional intriguing questions that should be answered to better understand the process of Fe^2+^ oxidation and the reactive oxygen species detoxification thought to be associated with the function of bacterioferritin molecules.

From the perspective of mobilizing Fe^3+^ stored in Bfr for its subsequent incorporation in metabolism, it is of note that, in *P. aeruginosa*, this process requires bacterioferritin to bind the [2Fe-2S] containing Bfd to, thereby, reduce Fe^3+^ in the internal cavity of Bfr and mobilize Fe^2+^ to the cytosol [2,9]. Blocking the bacterioferritin-Bfd complex by deletion of the *bfd* gene (Δ*bfd*) causes an irreversible accumulation of Fe^2+^ in bacterioferritin [9], which leads to intracellular iron starvation, global metabolic dysregulation, and impaired biofilm maintenance [26,35]. Moreover, small molecule inhibitors of the bacterioferritin-Bfd complex have been demonstrated to penetrate the envelope of *P. aeruginosa*, bind bacterioferritin in the cytosol, inhibit mobilization of stored Fe^3+^, and elicit cell death in mature biofilms [27,36]. These investigations of the physiological and potential antibiofilm target roles of the bacterioferritin-Bfd complex were conducted assuming that bacterioferritin in *P. aeruginosa* existed solely as a 24-mer of BfrB subunits. Consequently, it is important to stress that the data presented here demonstrates that Bfd binds heterooligomeric Bfr exclusively at the surface formed by BfrB inter-subunit dimers. Furthermore, the strength of the association is minimally affected by the presence of FtnA inter-subunit dimers as indicated by the nearly invariant *K*_d_ values obtained with Bfr molecules consisting of 100% BfrB, ~55% BfrB, or BfrB_rec_ (Table 2). 

## Figures and Tables

**Table 1:** The proportion of FtnA and BfrB subunits in Bfr is affected by O_2_ availability.

Fraction from Source^TM^ 15Q	%FtnA in Bfr			
	21% O_2_	10% O_2_	2% O_2_	Anaerobic
1st peak ^a^	0			
2nd peak ^a^	10 ± 1			
shoulder ^a^	21 ± 0.6			
left ^b^		28 ± 1	30 ± 4	29 ± 1
center ^b^		38 ± 2	45 ± 6	47 ± 2
right ^b^		49 ± 3	59 ± 1	69 ± 2

^a^ Bfr elutes from as Source^TM^ 15Q column in two peaks and a shoulder (e.g., see Figure 3A). ^b^ Bfr elutes from a Source^TM^ 15Q column in a single broad peak (e.g., see Figure 4A); left, center, and right denote fractions from the left, center, and right portions of the peak.

**Table 2:** The association between Bfd and Bfr as determined by SPR.

%FtnA in Bfr	*K*_d_ (μM)	Immobilization Level (RU)	R_max_	Ref.
0 (21% O_2_)	4.7 ± 0.8	14,003	3430	This work
45 (2% O_2_)	4.3 ± 0.5	14,059	2169	This work
100 (FtnA_rec_)	-	9497	-	This work
0 (BfrB_rec_)	3.3 ± 0.5	-	-	[23]

## Data Availability

Not applicable.

## Supplementary Materials

The following supporting information can be downloaded at: https://www.mdpi.com/article/10.3390/biom12030366/s1, Figure S1: Bfr purified from *P. aeruginosa* PAO1 is assembled from 24 subunits; Figure S2: Bfr isolated from PAO1 cells cultured in 2% O_2_ is a mix of 24-mer heterooligomeric Bfr constituted of FtnA and BfrB subunits; Figure S3: Bfr isolated from *P. aeruginosa* PAO1 cells cultured under anaerobic conditions is a mix of 24-mer heterooligomeric Bfr constituted of FtnA and BfrB subunits; Table S1: Proteins present in the excised gel band from a native gel lane loaded with Δ*ftnA* lysate solution obtained from cells cultured aerobically; Table S2: Proteins present in the excised gel band from a native gel lane loaded with PAO1 lysate solution obtained from cells cultured aerobically. Table S3: Proteins present in the excised gel band from a native gel lane loaded with Δ*bfrB* lysate solution obtained from cells cultured anaerobically.
