# Supplementary material for: Pseudomonas aeruginosa Bacterioferritin Is Assembled from FtnA and BfrB Subunits with the Relative Proportions Dependent on the Environmental Oxygen Availability
Source: Biomolecules. 2022 Feb 25;12(3):366. doi: 10.3390/biom12030366 (PMC8945002; doi:10.3390/biom12030366)
Supplement: Supplementary file 1 [file biomolecules-12-00366-s001.zip › biomolecules-1602477-supplementary.pdf]

Supplementary Information

Pseudomonas aeruginosa Bacterioferritin is Assembled from FtnA and BfrB Subunits with the Relative Proportions Dependent on the Environmental Oxygen Availability

Huili Yao[1], Anabel Soldano[1], Leo Fontenot[1], Fabrizio Donnarumma[1], Scott Lovell[2], Josephine R Chandler[3], and Mario Rivera[1,*]

[1] Department of Chemistry, Louisiana State University, 232 Choppin Hall, Baton Rouge, LA, 70803, USA; hyao@lsu.edu (H. Y.); asoldano@lsu.edu (A. S.); lfont39@lsu.edu (L. F.); fabrizio@lsu.edu (F. D.)
[2] Protein Structure and X-ray Crystallography Laboratory, University of Kansas, 2030 Becker Dr., Lawrence, KS 66047, USA; swlovell@ku.edu
[3] Department of Molecular Biosciences, University of Kansas, 1220 Sunnyside Ave., Lawrence, KS, 66045, USA; jrchandler@ku.edu
* Correspondence: mrivera@lsu.edu

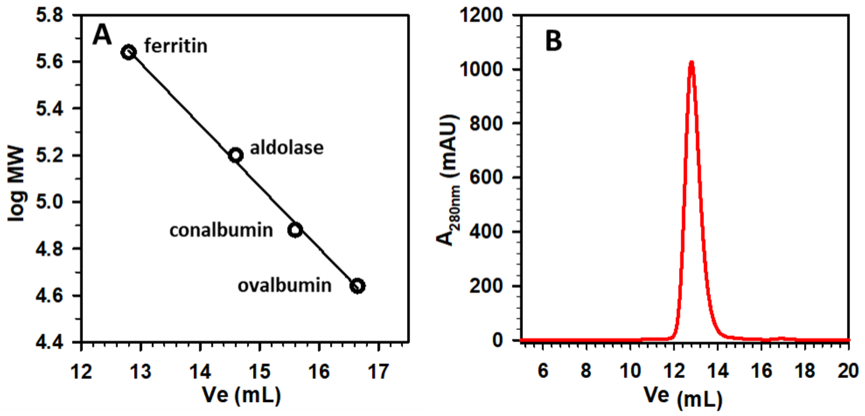

C

Protein	Ve (mL)
Standard ferritin	12.80
Bfr (21% O_2)	12.80
Bfr (10% O_2)	12.82
Bfr (2% O_2)	12.82
Bfr (anaerobic)	12.84

Figure S1. Bfr purified from *P. aeruginosa* PAO1 is assembled from 24 subunits. (**A**) Calibration curve obtained using the elution volume (V_e) of 24-mer ferritin (440 kDa), aldolase (158 kDa), conalbumin (75 kDa), and ovalbumin (44 kDa), loaded onto a Superdex200 Increase (10 × 300GL) column equilibrated and eluted with 100 mM potassium phosphate, 1 mM TCEP (pH 7.6). (**B**) Representative chromatogram obtained when Bfr isolated from PAO1 cells cultured in 21% O_2 was loaded onto the same Superdex200 Increase column equilibrated and eluted with 100 mM potassium phosphate, 1 mM TCEP (pH 7.6). (**C**) Comparing the V_e values of Bfr isolated from PAO1 cells cultured aerobically (21% O2), micro aerobically (10% or 2% O_2), and anaerobically, indicates that the isolated Bfr molecules are 24-mers.

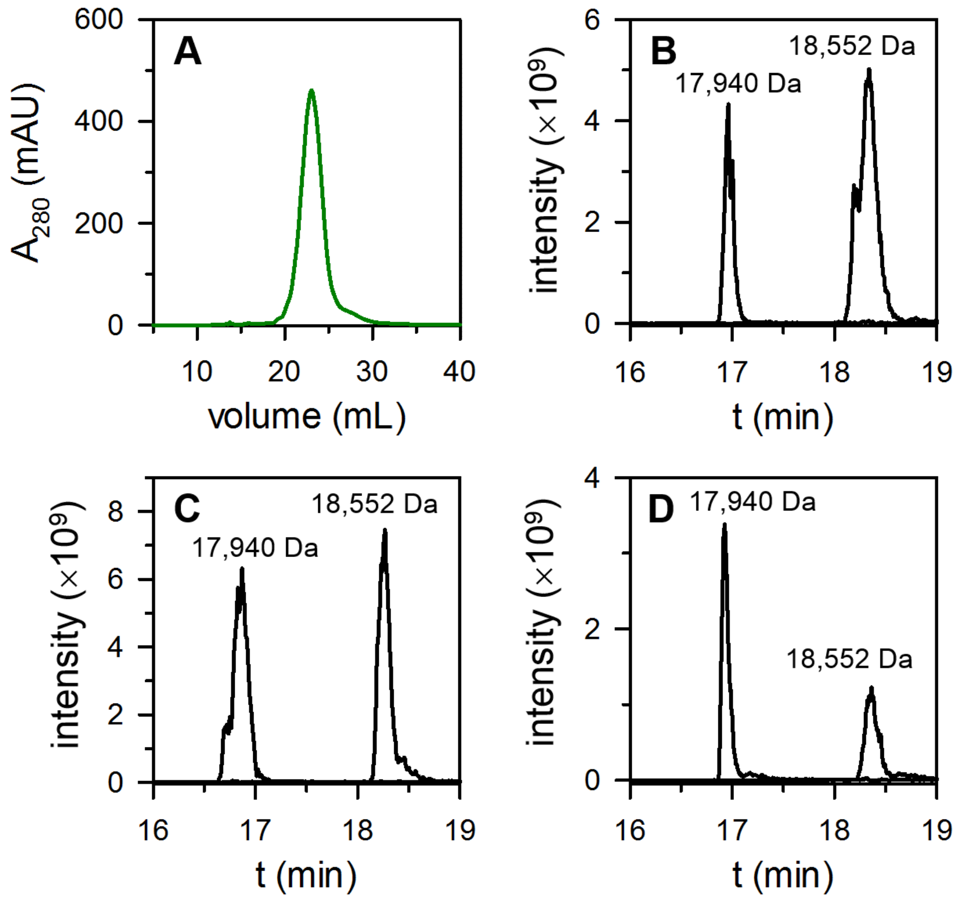

Figure S2. Bfr isolated from *P. aeruginosa* PAO1 cells cultured in 2% O_2 is a mix of 24-mer heterooligomeric Bfr constituted of FtnA and BfrB subunits. (**A**) Pure Bfr injected on a Source^{TM} 15Q column elutes in a single peak. The extracted ion chromatograms (EICs) obtained from LC-MS analysis of protein eluting from the Source^{TM} 15Q column in the (**B**) left, (**C**) center, and (**D**) right sections of the peak show the presence of FtnA (17,940 Da) and BfrB (18,552 Da) subunits. The content of FtnA and BfrB subunits in Bfr was estimated from the corresponding integrated peak areas in the EIC and the values are presented in Table 1.

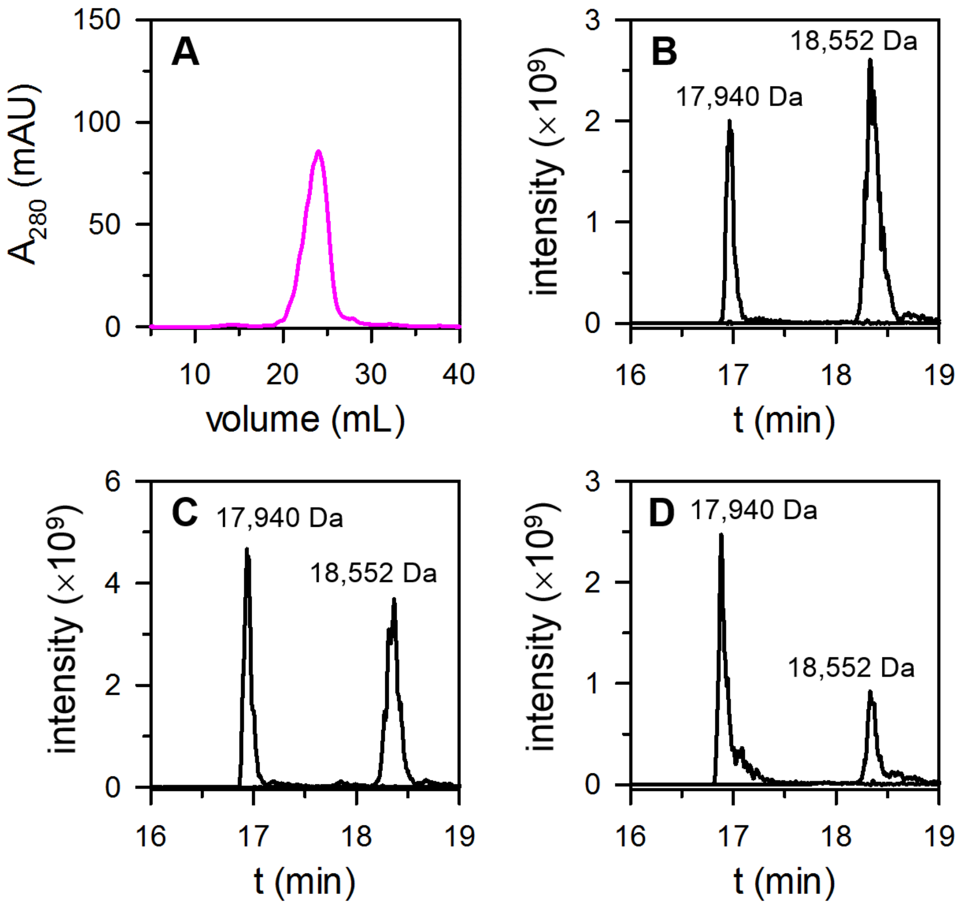

Figure S3. Bfr isolated from *P. aeruginosa* PAO1 cells cultured under anaerobic conditions is a mix of 24-mer heterooligomeric Bfr constituted of FtnA and BfrB subunits. (**A**) Pure Bfr injected on a Source™ 15Q column elutes in one peak. The extracted ion chromatograms (EICs) obtained from LC-MS analysis of protein eluting from the Source™ 15Q column in the (**B**) left, (**C**) center, and (**D**) right sections of the peak show the presence of FtnA (17,940 Da) and BfrB (18,552 Da) subunits. The content of FtnA and BfrB subunits in Bfr was estimated from the corresponding integrated peak areas in the EIC and the values are presented in Table 1.

Table S1. Proteins present in the excised gel band from a native gel lane loaded with Δ*ftnA* lysate solution obtained from cells cultured aerobically. The proteins presented in the table all Sum PEP Score greater than 40 and are sorted from higher score to lower score.

Accession	Description	Sum PEP Score	Coverage [%]	# Peptides	# PSMs	# Unique Peptides	# AAs	MW [kDa]
PA4812 ref	gi|308198350| formate dehydrogenase-O, major subunit	265.93	60	44	78	44	1025	113.9
PA4735 ref	gi|15599929| hypothetical protein	197.53	42	36	97	36	1088	118.5
PA4385 ref	gi|15599581| GroEL protein	170.35	60	26	199	26	547	57.1
PA5307 ref	gi|15600500| hypothetical protein	168.48	55	30	82	30	855	92.9
PA5146 ref	gi|15600339| hypothetical protein	158.19	49	32	83	32	750	80.8
PA4423 ref	gi|15599619| conserved hypothetical protein	145.70	53	20	70	20	604	65.6
PA5368 ref	gi|15600561| membrane protein component of ABC phosphate transporter	143.46	51	24	81	24	677	73.8
PA3922 ref	gi|15599117| conserved hypothetical protein	141.67	57	21	78	21	455	51.2
PA2815 ref	gi|15598011| probable acyl-CoA dehydrogenase	136.14	47	25	71	25	815	88.7
PA3724 ref	gi|15598919| elastase LasB	135.19	52	20	76	20	498	53.7
PA1342 ref	gi|15596539| AatJ	130.21	73	20	123	20	302	33.0
PA3848 ref	gi|15599043| hypothetical protein	127.48	48	20	76	20	451	50.5
PA2249 ref	gi|15597445| branched-chain alpha-keto acid dehydrogenase (lipoamide component)	126.90	53	18	75	18	428	45.7
PA4489 ref	gi|15599685| MagD	126.13	31	30	71	30	1516	167.3
PA1587 ref	gi|15596784| dihydrolipoamide dehydrogenase Lpd	121.48	45	19	68	19	478	50.1
PA3531 ref	gi|15598727| bacterioferritin	109.92	83	11	73	11	158	18.5
PA1586 ref	gi|15596783| dihydrolipoamide succinyltransferase (E2 subunit)	98.24	53	17	53	17	409	42.9
PA2999 ref	gi|15598195| Na+-translocating NADH:ubiquinone oxidoreductase subunit Nrq1	97.34	59	20	46	20	445	48.1
PA3836 ref	gi|15599031| hypothetical protein	95.23	58	14	41	14	325	34.2
PA0459 ref	gi|15595656| probable ClpA/B protease ATP binding subunit	90.44	32	21	50	19	850	94.1
PA4786 ref	gi|15599980| probable short-chain dehydrogenase	88.76	64	18	52	18	451	47.2
PA5554 ref	gi|15600747| ATP synthase beta chain	86.72	50	15	42	15	458	49.5
PA5435 ref	gi|15600628| probable transcarboxylase subunit	85.86	41	15	45	15	607	66.1
PA4277 ref	gi|15599473| elongation factor Tu	83.98	46	13	45	13	397	43.3
PA5366 ref	gi|15600559| ATP-binding component of ABC phosphate transporter	83.28	70	18	54	18	277	31.0
PA5367 ref	gi|15600560| membrane protein component of ABC phosphate transporter	80.94	37	16	52	16	558	61.2
PA0143 ref	gi|119167454| purine nucleosidase Nuh	80.08	43	8	34	8	350	37.5
PA4022 ref	gi|15599217| hydrazone dehydrogenase, HdhA	76.37	31	11	32	11	506	54.9
PA2338 ref	gi|15597534| probable binding protein component of ABC maltose/mannitol transporter	75.10	47	15	40	15	436	47.3
PA5556 ref	gi|15600749| ATP synthase alpha chain	73.51	28	14	38	14	514	55.4
PA1327 ref	gi|15596524| probable protease	72.35	31	19	44	19	656	72.5
PA1585 ref	gi|15596782| 2-oxoglutarate dehydrogenase (E1 subunit)	72.14	28	17	41	17	943	105.8
PA4491 ref	gi|15599687| MagB	71.86	40	16	36	16	589	64.2
PA5369 ref	gi|15600562| phosphate ABC transporter, periplasmic phosphate-binding protein, PstS	71.11	58	12	34	12	323	34.5
PA3313 ref	gi|15598509| hypothetical protein	69.45	53	14	38	14	335	36.5
PA1940 ref	gi|15597136| hypothetical protein	67.54	49	14	33	14	379	42.7
PA0946 ref	gi|15596143| hypothetical protein	62.52	59	10	29	10	345	36.8
PA5143 ref	gi|15600336| imidazoleglycerol-phosphate dehydratase	61.38	59	9	36	9	197	21.9
PA3187 ref	gi|15598383| probable ATP-binding component of ABC transporter	61.29	42	13	39	13	386	42.2
PA5119 ref	gi|15600312| glutamine synthetase	60.22	35	11	38	11	469	51.9
PA3081 ref	gi|15598277| conserved hypothetical protein	57.70	52	14	33	14	455	50.6
PA4394 ref	gi|15599590| conserved hypothetical protein	56.62	32	7	28	7	278	29.8
PA4825 ref	gi|15600018| Mg(2+) transport ATPase, P-type 2	56.24	17	14	36	14	903	100.0
PA0070 ref	gi|15595268| TagQ1	53.90	35	7	20	7	304	31.7
PA5045 ref	gi|15600238| penicillin-binding protein 1A	53.36	30	19	36	19	822	91.1
PA1807 ref	gi|15597004| NppD	53.33	36	12	29	12	536	59.0
PA1984 ref	gi|15597180| NAD+ dependent aldehyde dehydrogenase ExaC	52.37	24	8	24	8	506	54.9
PA5418 ref	gi|15600611| sarcosine oxidase alpha subunit	51.22	18	12	24	12	1005	109.3
PA4785 ref	gi|15599979| probable acyl-CoA thiolase	50.71	41	12	35	12	425	45.9
PA2953 ref	gi|15598149| electron transfer flavoprotein-ubiquinone oxidoreductase	49.82	34	14	30	14	551	59.9
PA2542 ref	gi|15597738| conserved hypothetical protein	48.38	17	13	32	13	1221	130.5
PA3770 ref	gi|15598965| inosine-5'-monophosphate dehydrogenase	48.22	34	10	26	10	489	51.7
PA3079 ref	gi|15598275| hypothetical protein	47.24	23	13	28	13	793	87.3
PA3816 ref	gi|15599011| O-acetylserine synthase	46.13	40	8	24	8	258	27.9
PA3999 ref	gi|15599194| D-ala-D-ala-carboxypeptidase	46.01	32	9	23	9	386	42.4
PA5237 ref	gi|15600430| conserved hypothetical protein	45.74	43	12	28	12	488	54.6
PA5076 ref	gi|15600269| probable binding protein component of ABC transporter	45.25	48	11	24	11	266	29.7
PA3026 ref	gi|15598222| conserved hypothetical protein	44.68	30	11	26	11	531	58
PA4639 ref	gi|15599835| hypothetical protein	44.16	54	8	46	8	195	20.7
PA3262 ref	gi|15598458| probable peptidyl-prolyl cis-trans isomerase, FkbP-type	43.98	32	7	24	7	253	26.8
PA3716 ref	gi|15598911| hypothetical protein	43.91	34	11	23	11	568	60.5
PA1941 ref	gi|15597137| hypothetical protein	43.30	24	13	26	13	631	70.9
PA1171 ref	gi|15596368| SltB2	43.08	30	10	22	10	398	42.7
PA4454 ref	gi|15599650| conserved hypothetical protein	42.94	73	8	21	8	157	16.5
PA5555 ref	gi|15600748| ATP synthase gamma chain	41.97	48	9	21	9	286	31.5
PA5558 ref	gi|15600751| ATP synthase B chain	41.22	46	6	24	6	156	16.9
PA4100 ref	gi|15599295| probable dehydrogenase	41.11	31	12	24	12	559	60.3
PA2973 ref	gi|15598169| probable peptidase	40.65	34	7	15	7	326	36
PA5312 ref	gi|15600505| Aldehyde dehydrogenase	40.44	27	8	19	8	497	53.1

Table S2. Proteins present in the excised gel band from a native gel lane loaded with PAO1 lysate solution obtained from cells cultured aerobically. The proteins presented in the table have Sum PEP Score greater than 40 and are sorted from higher score to lower score.

Description	Sum PEP Score	Coverage [%]	# Peptides	# PSMs	# Unique Peptides	# AAs	MW [kDa]
gi|15599581| GroEL protein	353.22	78	39	420	39	547	57.1
gi|15598011| probable acyl-CoA dehydrogenase	237.25	62	35	111	35	815	88.7
gi|308198350| formate dehydrogenase-O, major subunit	232.49	59	43	67	43	1025	113.9
gi|15599929| hypothetical protein	182.17	42	36	99	36	1088	118.5
gi|15599619| conserved hypothetical protein	175.77	51	21	72	21	604	65.6
gi|15599117| conserved hypothetical protein	168.30	69	25	77	25	455	51.2
gi|15596539| AatJ	156.01	76	24	106	24	302	33
gi|15596784| dihydrolipoamide dehydrogenase Lpd	151.53	43	20	68	20	478	50.1
gi|15600339| hypothetical protein	146.59	49	29	74	29	750	80.8
gi|15600500| hypothetical protein	141.69	45	27	72	27	855	92.9
gi|15600561| membrane protein component of ABC phosphate transporter	139.99	50	22	68	22	677	73.8
gi|15599685| MagD	138.86	33	30	72	30	1516	167.3
gi|15598919| elastase LasB	131.91	53	22	70	22	498	53.7
gi|15597445| branched-chain alpha-keto acid dehydrogenase (lipoamide component)	130.98	48	18	58	18	428	45.7
gi|15599043| hypothetical protein	120.83	48	18	68	18	451	50.5
gi|15599980| probable short-chain dehydrogenase	120.61	55	21	61	21	451	47.2
gi|15599031| hypothetical protein	117.90	60	17	55	17	325	34.2
gi|15600560| membrane protein component of ABC phosphate transporter	112.32	34	17	60	17	558	61.2
gi|15596524| probable protease	97.79	35	20	54	20	656	72.5
gi|15598965| inosine-5'-monophosphate dehydrogenase	93.09	54	17	51	17	489	51.7
gi|15600312| glutamine synthetase	92.17	41	16	51	16	469	51.9
gi|15599431| bacterial ferritin	91.11	84	11	38	11	154	17.9
gi|15600559| ATP-binding component of ABC phosphate transporter	89.23	71	18	52	18	277	31
gi|15596783| dihydrolipoamide succinyltransferase (E2 subunit)	84.07	41	13	37	13	409	42.9
gi|15599217| hydrazone dehydrogenase, HdhA	83.86	36	12	32	12	506	54.9
gi|15599687| MagB	79.99	36	19	42	19	589	64.2
gi|15598509| hypothetical protein	79.27	53	13	38	13	335	36.5
gi|15600749| ATP synthase alpha chain	78.74	28	13	38	13	514	55.4
gi|15598727| bacterioferritin	78.18	85	11	59	11	158	18.5
gi|15600238| penicillin-binding protein 1A	78.08	27	19	48	19	822	91.1
gi|15600747| ATP synthase beta chain	75.36	39	11	34	11	458	49.5
gi|15598195| Na+-translocating NADH:ubiquinone oxidoreductase subunit Nrq1	71.55	49	15	32	15	445	48.1
gi|15597136| hypothetical protein	70.41	47	14	38	14	379	42.7
gi|15599888| ketol-acid reductoisomerase	68.11	38	9	30	9	338	36.4
gi|15600562| phosphate ABC transporter, periplasmic phosphate-binding protein, PstS	67.72	59	12	32	12	323	34.5
gi|15597534| probable binding protein component of ABC maltose/mannitol transporter	66.28	32	13	35	13	436	47.3
gi|15597004| NppD	61.03	30	13	27	13	536	59
gi|15600269| probable binding protein component of ABC transporter	58.11	43	11	29	11	266	29.7
gi|15596143| hypothetical protein	57.72	62	11	28	11	345	36.8
gi|15598222| conserved hypothetical protein	56.64	36	13	33	13	531	58
gi|15600018| Mg(2+) transport ATPase, P-type 2	56.51	18	15	35	15	903	100
gi|15600628| probable transcarboxylase subunit	55.32	30	15	38	15	607	66.1
gi|15599590| conserved hypothetical protein	54.98	33	7	26	7	278	29.8
gi|15597180| NAD+ dependent aldehyde dehydrogenase ExaC	54.80	29	9	22	9	506	54.9
gi|15596782| 2-oxoglutarate dehydrogenase (E1 subunit)	54.78	23	14	35	14	943	105.8
gi|119167454| purine nucleosidase Nuh	54.54	37	7	24	7	350	37.5
gi|15600753| ATP synthase A chain	50.88	22	5	16	5	289	31.9
gi|15595656| probable ClpA/B protease ATP binding subunit	50.83	20	12	26	11	850	94.1
gi|15598458| probable peptidyl-prolyl cis-trans isomerase, FkbP-type	50.62	29	7	24	7	253	26.8
gi|15599473| elongation factor Tu	50.41	39	10	27	10	397	43.3
gi|15600611| sarcosine oxidase alpha subunit	49.95	15	10	22	10	1005	109.3
gi|15599194| D-ala-D-ala-carboxypeptidase	49.73	29	10	22	10	386	42.4
gi|15596786| succinyl-CoA synthetase alpha chain	49.08	52	9	30	9	295	30.2
gi|15596785| succinyl-CoA synthetase beta chain	45.93	37	11	24	11	388	41.5
gi|15599835| hypothetical protein	45.71	59	8	30	8	195	20.7
gi|15595599| aspartate carbamoyltransferase	44.25	37	8	24	8	334	36.6
gi|15600336| imidazoleglycerol-phosphate dehydratase	43.79	55	8	24	8	197	21.9
gi|15600365| ornithine carbamoyltransferase, catabolic	42.34	32	8	24	8	336	38.1
gi|15600430| conserved hypothetical protein	42.18	38	11	26	11	488	54.6
gi|15599011| O-acetylserine synthase	41.92	40	8	20	8	258	27.9
gi|15595268| TagQ1	40.92	29	6	16	6	304	31.7
gi|15595218| T4P secretin-associated protein TsaP	40.89	35	8	20	8	341	37.6
gi|15598277| conserved hypothetical protein	40.23	32	9	20	9	455	50.6
gi|15599295| probable dehydrogenase	40.00	28	10	20	10	559	60.3

Table S3. Proteins present in the excised gel band from a native gel lane loaded with Δ*bfrB* lysate solution obtained from cells cultured anaerobically. The proteins presented in the table have Sum PEP Score greater than 40 and are sorted from higher score to lower score.

Accession	Description	Sum PEP Score	Coverage [%]	# Peptides	# PSMs	# Unique Peptides	# AAs	MW [kDa]
PA3875 ref	gi|15599070| respiratory nitrate reductase alpha chain	309.16	62	52	228	52	1261	140.9
PA0519 ref	gi|15595716| nitrite reductase precursor	218.53	64	33	175	33	568	62.6
PA3068 ref	gi|15598264| NAD-dependent glutamate dehydrogenase	187.21	33	45	115	45	1620	182.5
PA3874 ref	gi|15599069| respiratory nitrate reductase beta chain	165.53	65	34	134	34	513	58.1
PA4270 ref	gi|15599466| DNA-directed RNA polymerase beta chain	157.02	36	38	99	38	1357	150.7
PA0459 ref	gi|15595656| probable ClpA/B protease ATP binding subunit	156.87	50	31	88	29	850	94.1
PA4269 ref	gi|15599465| DNA-directed RNA polymerase beta* chain	131.45	33	36	92	36	1399	154.3
PA1587 ref	gi|15596784| dihydrolipoamide dehydrogenase Lpd	122.82	44	18	74	18	478	50.1
PA4235 ref	gi|15599431| bacterial ferritin	107.69	84	11	109	11	154	17.9
PA1174 ref	gi|15596371| periplasmic nitrate reductase protein NapA	85.11	37	23	58	23	829	92.9
PA3247 ref	gi|15598443| hypothetical protein	72.90	48	18	46	18	429	46.6
PA5505 ref	gi|15600698| probable TonB-dependent receptor	72.56	57	14	42	14	260	28.0
PA5167 ref	gi|15600360| DctP	70.19	45	11	43	11	331	37.0
PA5078 ref	gi|15600271| OpgG	61.72	36	16	39	16	525	59.4
PA3922 ref	gi|15599117| conserved hypothetical protein	61.50	49	11	34	11	455	51.2
PA3915 ref	gi|15599110| molybdopterin biosynthetic protein B1	59.59	76	9	22	9	185	19.7
PA3299 ref	gi|15598495| long-chain-fatty-acid--CoA ligase	58.82	37	12	34	12	562	61.6
PA0300 ref	gi|15595497| polyamine transport protein	56.39	54	13	51	13	367	40.6
PA3313 ref	gi|15598509| hypothetical protein	55.49	50	13	37	13	335	36.5
PA0265 ref	gi|15595462| glutaric semialdehyde dehydrogenase	55.37	37	10	24	10	483	51.6
PA2540 ref	gi|15597736| conserved hypothetical protein	53.21	39	13	28	13	586	65.4
PA4238 ref	gi|15599434| DNA-directed RNA polymerase alpha chain	51.94	34	11	32	11	333	36.6
PA4496 ref	gi|15599692| probable binding protein component of ABC transporter	50.05	34	12	36	9	537	60.0
PA1337 ref	gi|15596534| glutaminase-asparaginase	49.57	36	9	34	9	362	38.6
PA1094 ref	gi|15596291| flagellar capping protein FliD	49.24	40	11	29	11	474	49.4
PA2976 ref	gi|15598172| ribonuclease E	49.20	15	11	26	11	1057	117.4
PA5378 ref	gi|15600571| CbcX	48.68	43	11	24	11	312	34.4
PA4949 ref	gi|15600142| conserved hypothetical protein	47.07	29	8	22	8	502	51.5
PA3800 ref	gi|15598995| conserved hypothetical protein	45.80	55	10	30	10	380	40.4
PA0973 ref	gi|15596170| Peptidoglycan associated lipoprotein OprL precursor	44.17	39	5	25	5	168	17.9
PA5312 ref	gi|15600505| Aldehyde dehydrogenase	43.63	35	11	28	11	497	53.1
PA3236 ref	gi|15598432| BetX	41.49	42	8	24	8	285	31.1
